# Acknowledgment of Reviewers

**DOI:** 10.2188/jea.JE20190300

**Published:** 2019-12-05

The following scientists assisted the journal by reviewing manuscripts during the period November 1, 2018 to October 31, 2019.

We gratefully acknowledge their critical evaluation and assistance in selecting articles for publication.

**Table:** 

Abe, Sarah K.
Adachi, Hisashi
Ahn, Hyeong Sik
Akahane, Takemi
Akasaka, Hiroshi
Amagasa, Shiho
Amiri, Mohammadreza
Andersen, Lars L.
Ando, Emiko
Araki, Atsuko
Araki, Atsushi
Arima, Hisatomi
Arisawa, Kokichi
Asakura, Keiko
Baba, Sachiko
Baik, Inkyung
Bosetti, Cristina
Burns, Jane
Cai, Lin
Chen, De
Chen, Hongda
Chen, Ke-Xin
Chen, Wen
Cheng, Yawan
Cui, Jianfeng
Cuzick, Jack
Doi, Satomi
d’Orsi, Eleonora
Duncan, Laura J
Eguchi, Hisashi
Endoh, Kaori
Eriksson, Malin
Fujihara, Kazuya
Fujihara, Sho
Fujita, Yuki
Fujiyoshi, Akira
Fukasawa, Maiko
Fukazawa, Ryuji
Fukuda, Yoshiharu
Fukuhara, Masayo
Fukui, Keisuke
Gao, Yanhui
García-González, María Asunción
Gibo, Koichiro
Goto, Atsushi
Goto, Aya
Gülcan, Ferda
Hagimito, Akiko
Han, Changwoo
Hanazato, Masamichi
Harada, Kazuhiro
Haruyama, Yasuo
Hashimoto, Hideki
Hata, Jun
Hayashi, Kunihiko
Hayashi, Tomoshige
Higashi, Akane
Higashiyama, Aya
Hikichi, Hiroyuki
Hirata, Takumi
Hisamatsu, Takashi
Hishida, Asahi
Hiyoshi, Ayako
Hong, Gwi-Ryung Son
Hori, Ai
Hosono, Satoyo
Huang, Huang
Huang, Kuo-Chin
Iftner, Thomas
Ikeda, Ai
Ikeda, Nayu
Ikehara, Satoyo
Imai, Shinobu
Imamura, Fumiaki
Inada, Haruhiko
Inoue, Shigeru
Ishihara, Junko
Ishikawa, Hirofumi
Ishikawa, Shizukiyo
Ishikawa-takata, Kazuko
Ishimaru, Miho
Isumi, Aya
Itani, Osamu
Ito, Jun
Ito, Yuri
Iwagami, Masao
Iwasa, Hajime
Iwasaki, Masanori
Jee, Sun Ha
Jia, Wei-Ping
Jinnouchi, Hiroshige
Joshipura, Kaumudi J
Jun, Jae Kwan
Kabasawa, Keiko
Kachi, Yuko
Kakizaki, Masako
Kamada, Masamitsu
Kanamori, Satoru
Karakatsani, Anna
kashino, ikuko
Katanoda, Kota
Kato, Hirotaka
Kato, Tsuguhiko
Katsuya, Tomohiro
Kaufman, Jay
Kawakami, Koji
Kawano, Reo
Kawasaki, Ryo
Keenan, Katherine
Kelly, Helen
Kesselring, Randall
Kessler, Ronald
Kidokoro, Tetsuhiro
Kikuchi, Hiroyuki
Kim, Byung Jin
Kim, Hyeon Chang
Kingsbury, Ann
Kitano, Naomi
Ko, Eunyoung
Kohno, Kunie
Komatsu, Hirokazu
Kondo, Naoki
Konta, Tsuneo
Koyama, Toshihiro
Koyanagi, Yuriko
Kubota, Kiyoshi
Kubota, Yasuhiko
Kuchiba, Aya
Kumar, Santhosh
Kuriki, Kiyonori
Kurokawa, Naoyuki
Kurotani, Kayo
Kuwahara, Keisuke
Lage, Carolina
Lee, Christoph
Leventhal, John
Li, Lanjuan
Li, Ya-Min
Liao, Yiqun
Lin, Chunqing
Liu, Xiaoqiu
Liu, Xiaoyun
Machida, Youichi
Maruyama, Koutatsu
Matsui, Hiroki
Matsuo, Keitaro
Matsuoka, Yutaka
Matsuyama, Yusuke
Metoki, Hirohito
Mezawa, Hidetoshi
Michikawa, Takehiro
Miyagawa, Naoko
Miyake, Yoshihiro
Miyaki, Koichi
Mizuta, Akiko
Momma, Haruki
Morita, Ayako
Morita, Ichizo
Murakami, Keiko
Muraki, Isao
Murata, Chiyoe
Nagai, Masato
Nagao, Masanori
Nagasawa, Shin-ya
Nagata, Chie
Nagata, Masaharu
Nakahara, Shinji
Nakamura, Itaru
Nakamura, Mieko
Nakamura, Yasuyuki
Nakatochi, Masahiro
Nakayama, Masaharu
Nakayama, Shoji
Nakayama, Takeo
Nawa, Nobutoshi
Ng, Chris Fook Sheng
Ninomiya, Toshiharu
Nishi, Daisuke
Nishi, Nobuo
Nishiura, Hiroshi
Nofuji, Yu
Noma, Hisashi
Oba, Koji
Oba, Shino
Ogilvie, Gina S.
Oguma, Yuko
Ohara, Tomoyuki
Ohkusa, Yasushi
Ohsawa, Masaki
Oishi, Akiko
Okada, Rieko
Okamura, Tomonori
Okamura, Tsuyoshi
Okumura, Junko
Okumura, Yasuyuki
Onishi, Kazunari
Ono, Sachiko
Ota, Atsuhiko
Otsuka, Rei
Oze, Isao
Paganini-Hill, Annlia
Park, Boyoung
Peres, Marco A
Pitiphat, Waranuch
Robinson, David Taylor
Sairenchi, Toshimi
Saito, Eiko
Saito, Isao
Saito, Junko
Sakurai, Masaru
Sangthong, Rassamee
Sasai, Hiroyuki
Sasakabe, Tae
Sata, Fumihiro
Sato, Yukihiro
Satoh, Michihiro
Sawada, Norie
Sawada, Susumu
Schlaud, M
Seino, Satoshi
Shibata, Ai
Shimazaki, Yoshihiro
Shimazu, Taichi
Shimizu, Yuji
Shimokawa, Satoru
Shinozaki, Tomohiro
Shioda, Kayoko
Shirai, Kokoro
Shiraishi, Nao
Shivappa, Nitin
Shobugawa, Yugo
Sloan, Robert
Song, Minkyo
Subramanian, SV
Sugiyama, Hiromi
Sugiyama, kemmyo
Sugiyama, Takehiro
Suma, Shino
Sumi, Ayako
Sun, Xibin
Suominen, Anna L
Surkan, Pamela
Suzuki, Etsuji
Suzuki, Kohta
Suzuki, Koji
Suzuki, Yuka
Tabuchi, Takahiro
Tachimori, Hisateru
Takachi, Ribeka
Takahashi, Kunihiko
Takahashi, Kyo
Takahashi, Yoshimitsu
Takakura, Minoru
Takeshita, Tatsuya
Takeuchi, Ayano
Takeuchi, Kenji
Takezaki, Toshiro
Takura, Tomoyuki
Tanabe, Naohito
Tanaka, Junko
Tanaka, Keitaro
Tanaka, Shiro
Tani, Yukako
Tanigawa, Takeshi
Taniguchi, Yu
Tanikawa, Chizu
Tinker, Sarah
Togawa, Kayo
Tomata, Yasutake
Tomioka, Kimiko
Tomitaka, Shinichiro
Tondel, M
Toyokawa, Satoshi
Tsuboya, Toru
Tsuchihashi, Yuuki
Tsushita, Kazuyo
Uchida, Mitsuo
Ueda, Kayo
Uehara, Ritei
Umesawa, Mitsumasa
Urayama, Kevin
Utada, Mai
van der Waal, Daniëlle
Volken, Thomas
Wada, Keiko
Wang, Jung-Der
Watanabe, Mikio
Wei, J.-N.
Whitton, Clare
Yamagishi, Kazumasa
Yamaguchi, Miwa
Yamaji, Taiki
Yamakita, Mitsuya
Yamamoto, Yosuke
Yamamoto-Hanada, Kiwako
Yamato, Hiroshi
Yang, Bo
Yang, Hui-xia
Yang, Jiaxi
Yasuda, Shigemitsu
Yatsuya, Hiroshi
Yokomichi, Hiroshi
Yokoyama, Yoshie
Yokoyama, Yukari
Yokoyama, Yuri
Yoneoka, Daisuke
Yoshida, Daigo
Yoshida, Kazuki
Yoshimi, Itsuro
Yosuke, Inoue
Zaitsu, Masayoshi
Zhang, Yong-hong
Zhao, Genming

